# The unanswered question. When to undertake a maternity journey?

**DOI:** 10.1007/s40620-023-01865-9

**Published:** 2024-01-29

**Authors:** Dalia Younis, Rasha Samir Shemies

**Affiliations:** Mansoura Nephrology and Dialysis Unit, Mansoura University, Mansoura, Egypt

**Keywords:** Pregnancy, atypical hemolytic uremic syndrome, Chronic kidney disease

“I desperately wish to get pregnant, I am worried about the outcome of pregnancy, but my husband wants a baby, I have no other option.”

These are some of the words Mrs. K told us on her first visit to our clinic. Mrs. K is a G1P0 24-year-old woman. She had been diagnosed with atypical hemolytic uremic syndrome (aHUS) 5 years previously during her 1st pregnancy, that ended with a stillbirth. Unfortunately, her kidney function did not normalize afterwards and she was discharged from the hospital with a serum creatinine of 4 mg/dL. When Mrs. K first presented to our obstetric-nephrology clinic, her creatinine was 5.1 mg/dL, her proteinuria was 268 mg/day, and her blood pressure was 140/90 mm/Hg. She reported she had her intrauterine device removed three months previously, and expressed her desperate wish to have children, not only for her sake, but for the sake of her husband and, implicitly, of her couple.

The patient had been amenorrheic for one year on presentation and her chance of a spontaneous pregnancy was considered very low at that time. Several questions arose: what is the best approach for this woman to conceive in view of the underlying amenorrhea?

What is the best timing to plan for an assisted pregnancy, should this antedate or wait for a kidney transplant?

What is the future risk of aHUS recurrence particularly after transplantation? Historically, women with aHUS are unable to achieve a successful pregnancy due to the severity of their kidney disease, and for the few who did conceive, recurrence of aHUS was a significant risk [1].

There are many recent studies that recommend the use of eculizumab, a monoclonal anti-complement (C5) antibody, as a lifesaving therapy in this situation. Unfortunately, eculizumab is not available in our country.

Each step has uncertainties: doubts remain about maternal and fetal safety with fertility treatment in women with chronic kidney disease (CKD) as well as the potential detrimental impact on kidney function. Ovulation induction bears a risk of ovarian hyperstimulation syndrome. In vitro fertilization (IVF) requires high concentrations of FSH to be administered to the patient before oocyte retrieval. While the risks in CKD are not known, unfortunately, both IVF and CKD are independently associated with increased risk of preterm birth, low birth weight and perinatal mortality [2].

In the event of a pregnancy with kidney disease, accelerated loss of maternal kidney function is a possibility and initiation of dialysis may be needed. This represents a demanding task, requiring intensive treatment with up to six long-hour sessions a week [3]. The initiation of dialysis is always a hard decision, and, while we know that intensive dialysis is the best option in women on chronic hemodialysis, the right moment to start is not fully established.

Furthermore, pregnancy after kidney transplantation is not necessarily a peaceful journey given a diagnosis of aHUS. Post-transplant recurrence of aHUS has been encountered in 60% of patients, of whom 90% developed graft failure. One‐year graft survival was 32% for deceased donor transplants and 50% for living donor transplants [4].

We were not able to encourage the woman to conceive spontaneously and even were unable to recommend waiting for transplantation. We were powerless, but she had the power when she decided to conceive and not to wait for any reason. “Is it safe to start induction of ovulation?”: she asked.

The current literature allows only confirming the high risk of doing this and, even more specifically, of a new pregnancy.

The patient’s background of aHUS adds to the uncertainties as well as to the risks, not only for the pregnancy but also for the mother’s life.

We, as nephrologists, can only classify her possible future pregnancy as being at extremely high risk. However, no numbers are available, and no number will likely make a difference to a woman who desperately wants a baby. There is no absolute threshold for a “too high risk”, as for some women a 10% risk may be not acceptable, while for others a 10% chance would be enough to try.

The situation seems sensitive and complex; however, the patient made her decision, whatever the outcome, to go ahead and induce faster ovulation with fertility medications. Hopefully, she is still trying to do so.

We understand the extent of the social pressures surrounding her, even if she did not express them in specific clear words. The intense pressure put on some women comes from the panic that time is running out, and the frustration that must be felt when a woman is forever asked when she is going to have kids, when in fact she is desperate for a baby but is unable to conceive. The pressure that is exerted on women, mostly by their partners, to hurry into getting pregnant may sometimes be dangerous and even lead to further problems in the couple’s relationship. But to what extent should we support the heroic spirit of patients seeking maternity despite all fears?

Knowledge gaps are unlikely to all be filled, especially in rare diseases, even by large-scale global studies and consensus guidelines. What is true is that disparities and inequalities involving women living in less wealthy countries should come to an end: while not solving all problems, the availability of a highly effective treatment for aHUS would probably improve our patient’s outcome.

However, we will never be able to provide all our patients with life jackets before they sail on their challenging maternity journeys.

One thing, however, is achievable.

We can reassure our patients that we will always be at their side, even when we do not support their choices, and even when we hold their risks are “too high”.

## Data Availability

Our patient's complete medical record is available for viewing upon reasonable request.
